# Extending Molecular Subtyping in Alzheimer’s using Machine Learning Techniques and Alternative Splicing Data

**DOI:** 10.1002/alz.092588

**Published:** 2025-01-03

**Authors:** Lewis Edward Palmer

**Affiliations:** ^1^ Queen Mary University of London, London, London United Kingdom

## Abstract

**Background:**

Recent studies suggest the existence of distinct molecular subtypes within the AD patient cohort, characterized by distinct gene expression patterns in AD‐relevant genes and pathways. Understanding these putative subtypes may prove pivotal to the greater understanding of AD pathology and developing targeted therapeutic interventions. This study aims to extend existing research by employing omics data modalities beyond gene expression, gathered from the ROSMAP and MSBB Alzheimer’s studies.

**Method:**

Alternative splicing (AS) events were extracted from patient‐level RNAseq data. Dimensionality‐reduction and clustering techniques from machine learning were employed to detect the presence of robust clusters within the AD patient cohort. Bootstrapping of said techniques on random samples of patients and data was used to measure the robustness of the observed clustering.

**Results:**

Initial results indicate the presence of three robust clusters within the AD patient cohort, using AS data alone. This result echoes that of existing research that uses gene expression data from the same patient cohorts. This paves the way for further analysis of the AS events that separate the clusters, and their significance to previously described genes or pathways implicated in AD pathology.

**Conclusion:**

This work demonstrates the potential of AS in further describing putative molecular subtypes of AD and aims to highlight AS events in specific areas of interest that distinguish such subtypes from each other, further developing the emerging picture of the diverse molecular underpinnings of AD and hopefully contributing to more precise and effective medical interventions. Refining of the machine learning portion of the work to achieve the clearest delineation possible between clusters will facilitate a clearer understanding of the molecular signatures that characterise each patient cluster.

